# A Self-Aware and Scalable Solution for Efficient Mobile-Cloud Hybrid Robotics

**DOI:** 10.3389/frobt.2020.00102

**Published:** 2020-08-05

**Authors:** Aamir Akbar, Peter R. Lewis, Elizabeth Wanner

**Affiliations:** Aston Lab for Intelligent Collectives Engineering (ALICE), Computer Science, Aston University, Birmingham, United Kingdom

**Keywords:** mobile-cloud hybrid (MCH) computing, robotics, evolutionary algorithms, NSGA-II, multi-objective optimization (MOO), self-adaptive, self-aware, code offloading

## Abstract

Backed by the virtually unbounded resources of the cloud, battery-powered mobile robotics can also benefit from cloud computing, meeting the demands of even the most computationally and resource-intensive tasks. However, many existing mobile-cloud hybrid (MCH) robotic tasks are inefficient in terms of optimizing trade-offs between simultaneously conflicting objectives, such as minimizing both battery power consumption and network usage. To tackle this problem we propose a novel approach that can be used not only to instrument an MCH robotic task but also to search for its efficient configurations representing compromise solution between the objectives. We introduce a general-purpose MCH framework to measure, at runtime, how well the tasks meet these two objectives. The framework employs these efficient configurations to make decisions at runtime, which are based on: (1) changing of the environment (i.e., WiFi signal level variation), and (2) itself in a changing environment (i.e., actual observed packet loss in the network). Also, we introduce a novel search-based multi-objective optimization (MOO) algorithm, which works in two steps to search for efficient configurations of MCH applications. Analysis of our results shows that: (i) using self-adaptive and self-aware decisions, an MCH foraging task performed by a battery-powered robot can achieve better optimization in a changing environment than using static offloading or running the task only on the robot. However, a self-adaptive decision would fall behind when the change in the environment happens within the system. In such a case, a self-aware system can perform well, in terms of minimizing the two objectives. (ii) The Two-Step algorithm can search for better quality configurations for MCH robotic tasks of having a size from small to medium scale, in terms of the total number of their offloadable modules.

## 1. Introduction

In recent years, there has been a growing interest to bind virtual resources to low-power devices, such as mobile robots (Balan et al., 2002; Nakahara and Beder, 2018). To make robots virtually limitless in terms of processing power, energy and storage space, the integration of mobile applications with the cloud infrastructure (Armbrust et al., 2010) is often done. This interdisciplinary domain is called Mobile-Cloud Computing (MCC) (Huang, 2011).

A battery-powered mobile robot can be connected to the cloud infrastructure via a WiFi network. From the robot point of view: the decision to execute a computationally-intensive task locally can demand high battery power consumption, e.g., when an application is doing image processing. From a connection point of view: the decision to offload the computationally-intensive tasks remotely to the cloud can be affected by high network usage, e.g., when an application offloads to the cloud too often or a large amount of data is transferred between a robot and the cloud.

In MCC, an *efficiency trade-off* exists between the battery power consumption and network usage (Akbar and Lewis, 2017, 2018a). Due to which, the performance of Mobile-cloud hybrid (MCH) applications can be inefficient in terms of battery power consumption. Also, communication between a robot and the cloud can be inefficient in terms of data transmission cost along with battery power consumption by the transmitter chip of a robot. Therefore, achieving the objectives of minimum power consumption and network usage at the same time might not be possible. For example, minimizing network usage may prevent the objective of minimizing power consumption because the transceiver chip also uses power to send or receive data packets. To attain a set of optimal solutions, while not affecting the overall performance of an application, is considered one of the challenging areas in mobile-cloud computing (Ahmed et al., 2015).

We consider the effective partitioning of MCH applications as a multi-objective optimization (MOO) problem. In multi-objective optimization, there are several objectives to be optimized simultaneously, and typically the objectives are conflicting with each other. There exists a natural trade-off between the objectives, which creates a set of Pareto-optimal solutions (Deb, 2011), those that are not dominated by any possible other solution in the solution space. Therefore, we consider the following two objectives to optimize.

1. Minimize power consumption: the battery power consumption of an application on a robot, during one execution. We will measure it in joules (the unit of energy).
2. Minimize network usage: the data transferred between a mobile device and the cloud endpoint, during one execution. We will measure it in KBs.

To optimize the two objectives, we proposed a technique (Akbar and Lewis, 2017, 2018a), for Android-based smartphones, to find and apply the optimal configurations of an MCH application. We define a configuration as, *a valid mapping of all distinct and offloadable modules of an MCH application to a binary string*. The optimal configurations are the ones in which the application has minimal battery power consumption and minimal network usage per execution. To make the configurations for an MCH application, we first modularize the application: a set of collaborative code units called modules (i.e., classes or methods in Python) are made offloadable using an MCH application framework. The offloadable modules can be executed both locally on a robot and remotely on the cloud server. We then create a configuration set based on the number of offloadable modules.

In this paper, we extended our technique to be used for a MCH robotic environment. For evaluation, we employed a robotic task performed by a battery-powered and Raspberry Pi controlled robot. We highlight the three main contributions of this paper as follow.

1. Our MCH framework for robotics optimizes the efficiency trade-off between power consumption and network usage. We use offline profiling to search efficient configurations for MCH robotic tasks that are non-dominated by any other configurations.
2. Our MCH framework for robotics avoids the network latency. The framework is enabled with a self-adaptive and self-aware decision mechanism to switch between the efficient configurations at runtime. Therefore, optimizes the efficiency trade-off and avoids latency in the network.
3. We provide a solution to find approximate Pareto-optimal configurations for a small to a medium scale MCH applications for robotics in terms of the number of offloadable modules.

Analysis of our experimental results shows that: (1) using multi-objective optimization and code-offloading, the Pareto-optimal configurations can be searched and apply to the MCH robotic task. (2) Based on self-adaptivity and self-awareness, a system can achieve minimum power consumption and can also avoid network latency caused by packet loss due to interference, which reduces the network usage. However, the self-adaptive based decisions struggle when the packet loss is due to other factors, such as network congestion or link failure, in which case the network usage is high. In such a scenario, the self-aware based decision can achieve minimum power consumption and avoids latency caused by either low signal level or congestion, which minimizes the network usage. (3) A Two-Step search algorithm, which we developed, can produce better solutions compared to the current state-of-the-art NSGA-II algorithm (Deb et al., 2000) for a small to medium-scale MCH applications.

The rest of this paper is organized as follow. Section 2 discusses the related work. Section 3 discusses the required steps to modularize mobile applications and create their configurations. Section 4 describes the MCH application framework as well as the self-adaptive and self-aware decision mechanisms of the MCH application framework. Section 5 explains scalability of MCH applications. Section 6 explains the test-bed MCH application and the experimental study. Section 7 concludes the paper and the future work is discussed in section 8.

## 2. Related Work

The popularity of battery-powered robots has increased mainly because of their increased applications to numerous real-world problems. For example, unmanned search and rescue operations, automated manufacturing, self-driving vehicles and medical robots. Also, the applications created for mobile robots are intended to operate in extreme and high-risk conditions, e.g., seal a leak in a nuclear reactor or coordinate search and rescue missions; when natural disasters, such as earthquakes occur. Mobile robots are equipped with services like location awareness and sensors, which unlike humans can be deployed in dangerous sites with little risk. However, they are limited by their onboard resources (i.e., battery life). To address this problem, researchers have recently proposed cloud-enabled robotics technology. Cloud-enabled robotics can take advantages of cloud computing to minimize the battery power consumption of a mobile robot (Lin et al., 2015). As discussed in Saha and Dasgupta (2018), the integration of cloud computing with robotics have several advantages. We highlight them as follow.

1. The computationally-intensive tasks in cloud robotics, such as object recognition, computer vision and pattern matching are offloaded to the cloud for execution. Therefore, extending the battery life of mobile robots (Wan et al., 2016).
2. Cloud-enabled robots have virtually available high storage space to store data. Many applications in robotics, i.e., simultaneous localization and mapping (SLAM), generate a large amount of sensor data that is difficult to store with the limited onboard storage capacity on most robots (Hu et al., 2012).
3. Integrating cloud computing to robotics can enable robots to access big data, such as global maps for localization, object models that the robots might need for manipulation tasks as well as open-source algorithms and code (Kehoe et al., 2015).

### 2.1. Applications Partitioning

We are faced with a decision, when designing an MCH application, about what parts (modules) of the application should be executed locally and remotely. Therefore, the source code of an application is partitioned in such a way to identify the modules for local or remote executions. This can be achieved by using application partitioning algorithms (APAs) (Liu et al., 2015). When the partitioning is done during development time, the code units (i.e., classes/methods) are annotated and then using static analysis they are converted into offloadable modules by a converter. When the partitioning is done during execution time, a profile is used that decides on the fly which modules to execute on the cloud. Either type of partitioning algorithms aims to identify the most computationally-intensive modules for remote processing (Li et al., 2001; Gu et al., 2004).

One of the important aspects of APAs is the *partitioning granularity* attribute, which indicates the granularity level for partitioning computationally-intensive modules (Liu et al., 2015). Gu et al. (2003) framework for adaptive offloading of computationally-intensive modules is based on class-level granularity. Cuervo et al. (2010) used method-level partitioning of applications for their mobile cloud application framework. A thread-level (Chun et al., 2011) and object-level (Tilevich and Smaragdakis, 2002) partitioning has also been used to offload the computationally-intensive parts of applications to a remote computing server.

Previously, we have shown (Akbar and Lewis, 2018a) the partitioning of applications by modularizing the code into different levels of granularity using annotations during development. We highlighted the importance of granularity for efficient partitioning of the applications. In this paper, we will use a class-level, method-level and a hybrid-level (mix of class and method level) partitioning of applications that are developed using object-oriented programming paradigm in Python language.

### 2.2. Mobile-Cloud Hybrid Frameworks

The computation offloading from mobile devices to the cloud, inline with the Mobile Cloud Computing (MCC) paradigm (Huang, 2011; Shuja et al., 2016, 2018) is normally based on achieving one or more than one particular objective(s). For example, speeding up computation and lowering battery consumption in mobile devices as in CloneCloud (Chun et al., 2011), MAUI (Cuervo et al., 2010), or ThinkAir (Kosta et al., 2012). The increasing computing power and networking capabilities of mobile devices led to the consideration of Mobile Edge Clouds (MECs), which are formed by a neighborhood of devices (Brown, 2016; Rodrigues et al., 2017). The advantages of MECs include data locality, as data is usually produced at the edge, and of low network latencies afforded by local WiFi networks. Moreover, recently proposed a hybrid of MEC and cloud architectures are also considered in simulation frameworks like EdgeCloudSim (Suryavansh et al., 2019) or MobEmu (Ciobanu et al., 2019).

#### 2.2.1. Cloud-Enabled Robotic Frameworks

In the recent past, Chen et al. (2010) proposed Robot as a Service (RaaS) that is based on Service-Oriented Architecture (SOA) of cloud computing. RaaS facilitates the seamless integration of robot and embedded devices into Web and cloud computing environments. Backed by virtually unlimited resources of cloud computing, many computationally-intensive robotics and automation systems applications, such as robot navigation by performing SLAM in the cloud (Riazuelo et al., 2014) and next-view planning for object recognition (Oliveira and Isler, 2013) can be achieved. In Liu et al. (2014), the authors proposed a comprehensive distributed cloud-enabled robotics framework. Apart from combining cloud and the robot networks, they also provide additional security features in their framework. Moreover, in other recent works (Rahman et al., 2017; Chen et al., 2018; Hong et al., 2019) computation offloading in robotics has been used. Therefore, improving the performance of mobile robots as well as providing a cloud-based energy-efficient alternative.

#### 2.2.2. Self-Adaptive and Self-Aware Mobile-Cloud Hybrid Frameworks

Based on self-adaptation, Naqvi et al. (2016) proposed a multi-objective optimization framework called (*MAsCOT*), which employs Probabilistic Graphic Models (PGM) for a self-adaptive decision support for code offloading. Nakahara and Beder (2018) bi-objective optimization framework (*CoSMOS*) analyse each optimization parameters (energy consumption and execution time) separately using cost function and self-adaptive reinforcement. Furthermore, MCH applications can benefit from self-awareness (Preden et al., 2015). Dutt et al. (2016) applied self-aware decision mechanism to IoT hardware chips.

### 2.3. Insufficiencies in Available Approaches

To best of the author's knowledge, the previous works (Cuervo et al., 2010; Chun et al., 2011; Huang, 2011; Kosta et al., 2012; Shuja et al., 2016, 2018; Rahman et al., 2017; Chen et al., 2018; Hong et al., 2019), particularly in MCC, did not consider the multi-objective optimization approach to find an optimum balance between achieving the two objectives. Moreover, our framework allows for both offline profiling and online profiling. Offline profiling is to search for efficient configurations that optimize the trade-off between battery power consumption and network usage. Furthermore, we are the first to employ a self-adaptive (based on a change in the environment) and self-aware (based on a change within the system itself) decision mechanisms in our MCH framework for dynamic code-offloading to avoid network latency. By using online profiling, the framework switches between the efficient configurations to minimize battery power consumption, network usage and improve the performance of applications by avoiding network latency. Finally, unlike previous works, our approach is based on finding efficient configurations for MCH applications, which are then used by the applications during run-time. Therefore, reducing the computation overhead at run-time (i.e., running an optimizer on the mobile device). Doing so, we provide a scalable solution to finds approximate Pareto-optimal configurations for a small to medium scale MCH applications for in terms of the number of offloadable modules.

## 3. Efficient Modularization of Mobile Applications

The computationally-intensive tasks of mobile applications consumed battery power when executed. Such tasks of applications can be identified either during the development stage or after the developmental process is completed. We identify the code units (i.e., classes or methods) of MCH applications that have the computationally-intensive tasks at the code-level, using an MCH application framework. These code units are converted into offloadable modules using a technique called modularization. The offloadable modules can be executed both locally and remotely (i.e., on the cloud server endpoint). The remote execution of the modules can reduce the battery power consumption of an application with a trade-off of using the available network. We achieve this by executing MCH applications using a framework that works with a “configuration.” A range of different configurations for an application can be created, which is based on (1) the number of offloadable modules, (2) executing the modules across the two endpoints (i.e., a mobile device and the cloud), and (3) granularity-level of a module.

### 3.1. Granularity of Configurations

Granularity is the extent to which MCH applications can be partitioned into different modules. The partitioning can be done at different levels of granularity: class-level, method-level, object-level, thread-level, task-level, component-level. Since we will be using mobile applications that are developed using Object-Oriented programming (as we will discuss in the experimental study), therefore, we will consider method-level and class-level partitioning of applications. The computationally-intensive parts of an application are, therefore, divided into the resultant partitioned components; we called them modules. The modules are composed of code units or machine instructions. They might be fine-grained (i.e., methods of classes) or coarse-grained (i.e., classes of an application). The fine-grained modules comprise of a chunk of code that might be computationally-intensive or execute often during runtime of the application. In both cases, making the fine-grained modules offloadable and executing them remotely on the cloud can reduce power consumption. The coarse-grained modules are comprised of one or more fine-grained modules (methods).

### 3.2. Modular Configurations and Their Representation

A modular configuration (or simply a configuration) maps the offloadable modules to their execution points. It is created for executing an MCH application. To map the offloadable modules of an MCH application to a configuration, the number at which a module execute during the application runtime is assigned its position (or index) in the configuration. A module may execute multiple times during runtime, but its position in the configuration is determined by the number at which it executes for the first time.

We use a binary string to represent the configuration. Each digit (bit) of the string represents an offloadable module of the application. The state of a digit value signals the MCH framework to execute its representative module either on a mobile device endpoint (0) or on the cloud server endpoint (1). Based on the total number of modules and their granularity level, we can obtain different types of configurations, which are discussed as follow.

#### 3.2.1. Class-Level Configurations

**Figure 1:**
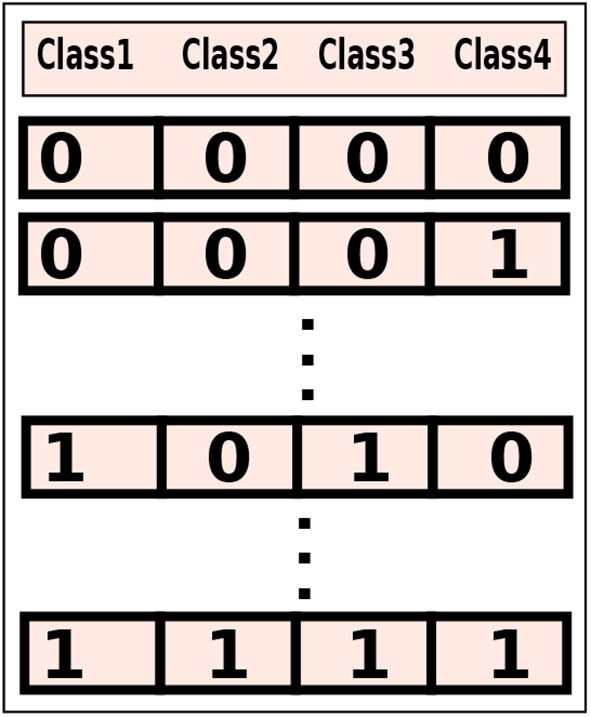
A class-level configuration set having all possible configurations for four offloadable classes of an application. The cardinality of the set is: 2^4^ = 16.

A class-level (coarse-grained) configuration executes the classes of an application, mapped to a binary string, on either a mobile device or on the cloud. For example, a configuration, 1010, which maps 4 classes of an application to a binary string. We can see that the first and third modules of this configuration will be executed on the mobile device (0) and the second and fourth modules will be executed to the cloud (1). For *n* = 4, a set containing all possible class-level configurations, as shown in Figure 1, can be obtained having total number of configurations equals to: 2^*n*^ = 16.

#### 3.2.2. Method-Level Configurations

**Figure 2:**
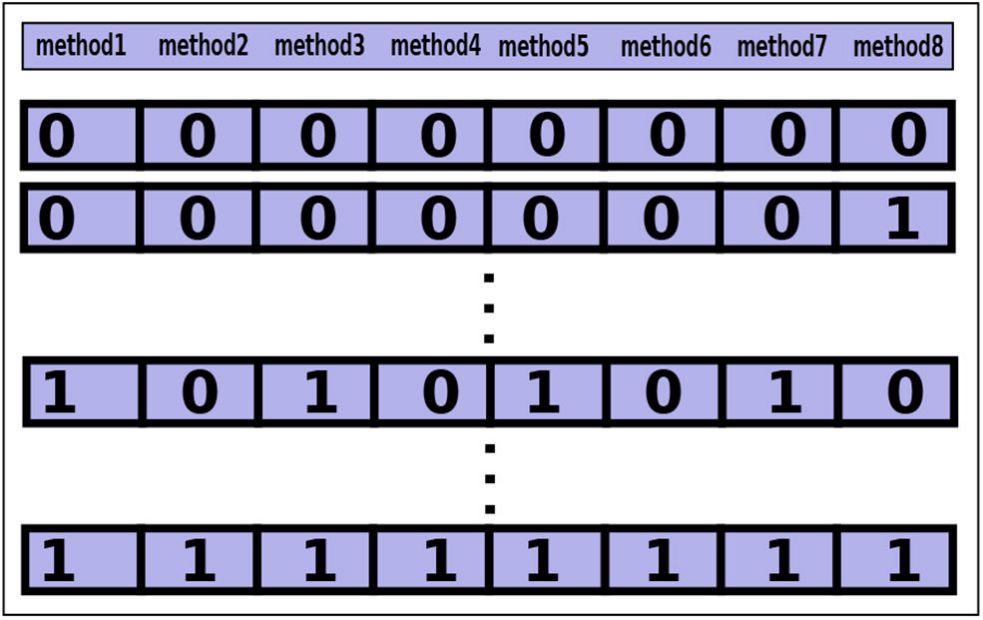
A method-level configuration set containing all possible combination of configurations for eight offloadable methods of an application.

Similar to the class-level configuration, a method-level configuration can also be created. In a method-level configuration, the binary string represents the methods where each digit of the string maps an offloadable method. For example, an arbitrary method-level configuration: 10101010, from a set shown in Figure 2. It maps eight (*n* = 8) modules of an application. To make the method-level configuration set, the cardinality of the set will be: 2^8^ = 256. Each configuration in the set is a valid combination of mapping the methods.

#### 3.2.3. Hybrid-Level Configurations

**Figure 3:**
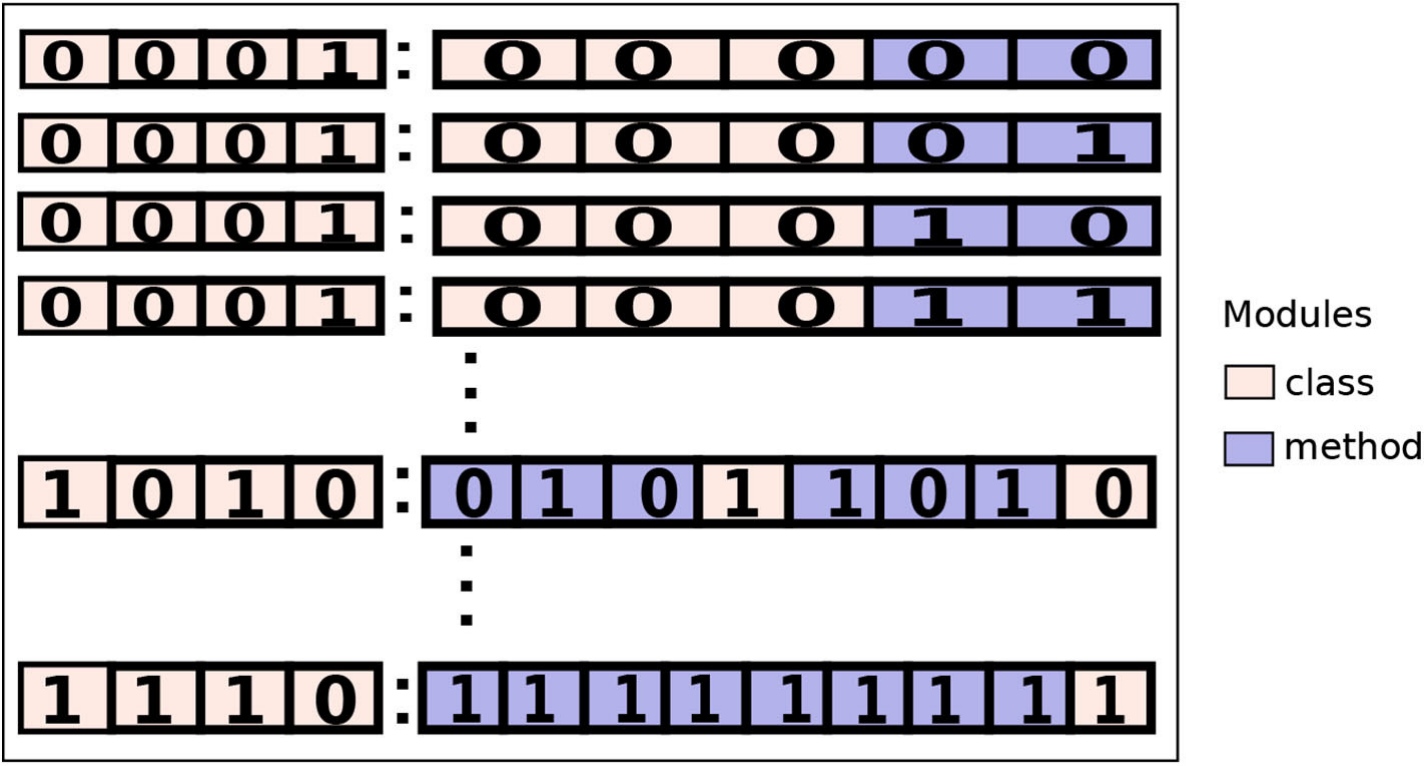
A hybrid-level configuration set containing all possible combination of configurations of an application.

A hybrid-level configuration has a mixed granularity. To create a hybrid-level configuration, we aim to map the offloadable modules in a mixed combination of selected methods and classes. A binary string can also represent a hybrid-level configuration, i.e., 1010:01011010, as shown in Figure 3. Unlike the class and method level configurations, the binary string is composed of two parts that are separated by a colon sign (:). The digits residing on its left side (first part) represent the offloadable classes and on its right (second part) is a combination of both classes and methods.

The state value of a digit in the first part signals whether the mapped class will be executed as a coarse-grained (0) or as a fine-grained (1). The state of a digit in the second part describes whether the mapped offloadable module (a class or a method) will be executed on the mobile (0) or the cloud (1). If a class is represented as fine-grained in the first part then its offloadable methods would be mapped. If it is represented as coarse-grained then it will be mapped.

For example, let's assume an arbitrary hybrid-level configuration: 1010:01011010. The first part of the configuration 1010, map four classes. Given that the first class has three methods and is mapped to be executed as fine-grained (1) all its three methods are mapped in the first three digits of the second part (010) where zero represents executing the method on the mobile device and one is for executing on the cloud. To make a hybrid-level configuration set, the cardinality of the set will depend on the total number of offloadable classes and methods of the application.

### 3.3. Collapsible Configurations

We define a collapsible configuration as a configuration that can be collapsed into the same or a different granularity level configuration. In other words, two or more configurations are called collapsible if they are of different or same granularity level, and they map the same modules to be executed on the same endpoints. Collapsible configurations are identical because during the runtime the same modules are executed on either a mobile device of the cloud server.

**Figure 4:**
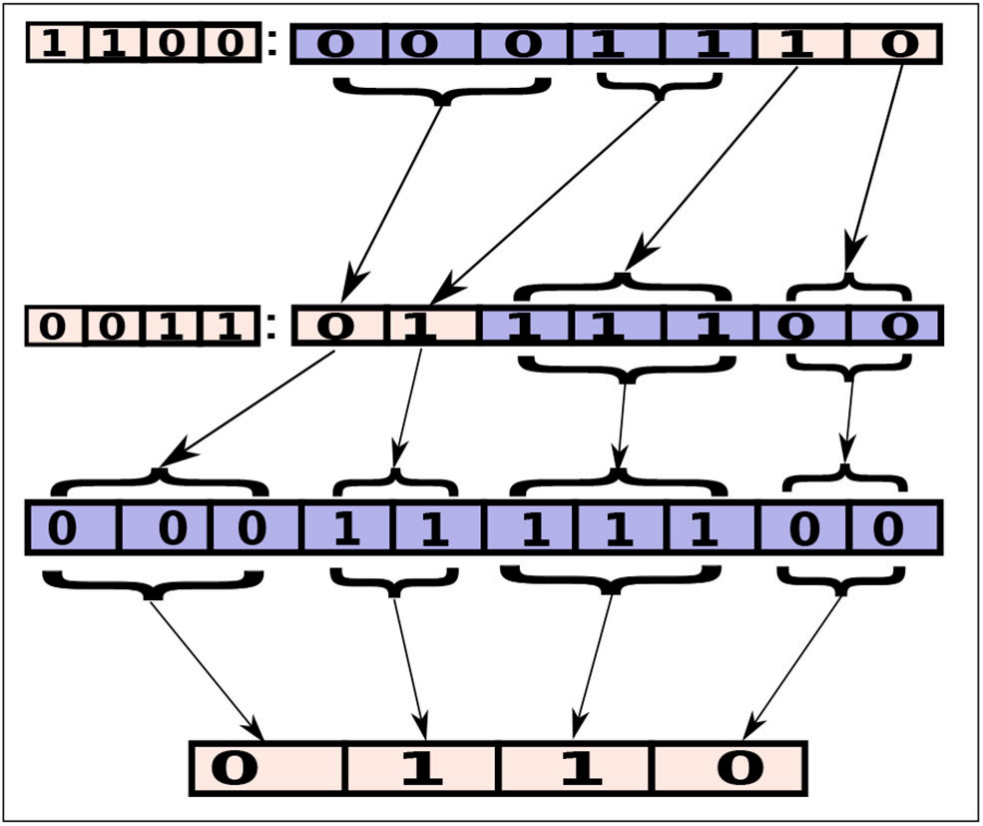
A set of four collapsible configurations. Each configuration is collapsible into another, where the execution endpoint of the offloadable modules are retained.

As shown in Figure 4, a hybrid-level configuration, 1100:0001110, is identical to another hybrid-level configuration (0011:0111100) a method-level configuration (0001111100) and a class-level configuration (0110). In first configuration (1100:0001110) the first three digits (representing three methods of a class) on the left side of the colon are zero, which is same as the class-level digit in the second configurations (0011:0111100). In these collapsible configurations, we have assumed four classes. First and third class have three offloadable methods and second and fourth class have two offloadable methods. These configurations will execute the same modules on the similar endpoints no matter what the configuration level is. Even though collapsible representations are equivalent from a configuration perspective, they may still lead to different battery power consumption and network usage, due to implementation details. For example, finding the mapped modules for a hybrid-level configuration will go through many steps of (if-else) statements. On the other hand, for a class-level configuration, it will take relatively less number of comparisons.

### 3.4. Effective Partitioning of MCH Applications

In an MCH application, the decision to execute the modules of the application either on a mobile device or on a cloud server is an NP-hard problem. Therefore, an optimal partition of the MCH application is desired based on one more objectives. Therefore, we consider the effective partitioning of an MCH application as a multi-objective optimization problem. In multi-objective optimization, there are several objectives to be optimized simultaneously, and typically the objectives are conflicting with each other. There exists a natural trade-off between objectives which creates a set of Pareto-optimal solutions (Deb, 2011), those that are not dominated by any possible other solution in the solution space. Therefore, optimizing the following two objectives, we might obtain Pareto-optimal configurations.

1. Minimize power consumption of an MCH application. We measured this as the number of joules consumed by the application during one execution.
2. Minimize network usage of an MCH application. We measured this as the data (KBs) sent and received between a mobile device and the cloud server, during one execution.

During the run-time of MCH applications, computationally-intensive tasks are offloaded to cloud servers for execution. One of the limitations of the offloading is that it often itself is computationally-intensive. For example, the cost of energy usage of transmitter chip and network usage are both associated with code offloading. Therefore, there exist a trade-off between the two objectives. To optimize the trade-off, we identify the offloadable modules of an applications and separate them from the modules that require mobile device hardware for execution. Each of the offloadable modules can either be executed on the mobile or cloud machine. Mathematically, given an *n* number of offloadable modules in a mobile application;

$$\begin{array}{l} M=\left\{m_{1},m_{2},m_{3},,,,,,m_{n}\right\}, \end{array}$$

(1) $$\begin{array}{l} \exists \alpha \left(m_{i}\right)=\left\{0,1\right\}\;\wedge \;\beta \left(m_{i}\right)=\left\{0,1\right\}\;|\;\alpha \left(m_{i}\right)+\beta \left(m_{i}\right)=1, \end{array}$$

(2) $$\begin{array}{l} min\left[\underset{m_{i}\in M}{\sum }\left(\alpha \left(m_{i}\right)\times C_{\alpha }\left(m_{i}\right)+\beta \left(m_{i}\right)\times C_{\beta }\left(m_{i}\right)\right)\right], \end{array}$$

$$\begin{array}{l} \forall m_{i}\in M, \end{array}$$

where a module, *m*_*i*_ ∈ *M*, can be executed either on a mobile device or on the cloud server. The binary functions α(*m*_*i*_) and β(*m*_*i*_) in Equation (1) are to determine whether a module *m*_*i*_ can be executed on the mobile device or on the cloud. The objective is to minimize the expression in Equation (2), which is to minimize the cost (i.e., the efficiency trade-off) associated with the module *m*_*i*_ for both end-points. As there are two end-points, the associated costs are of two different types. For the mobile device, the cost is *C*_α_(*m*_*i*_), and for the cloud server, it is *C*_β_(*m*_*i*_). Since we treat each of the two objectives independent of each other, the cost functions for a module, *m*_*i*_ ∈ *M*, return a *2-tuple*, i.e., (*P*_*i*_, δ_*i*_). Where *P*_*i*_ is the power consumption and δ_*i*_ is the network usage measured for a module, *m*_*i*_ ∈ *M*. Mathematically;

(3) $$\begin{array}{l} P_{\left(watts\right)}={P^{\text{C}}}_{\left(watts\right)}+{P^{\text{RF}}}_{\left(watts\right)}, \end{array}$$

(4) $$\begin{array}{l} \delta_{\left(Bytes\right)}={B^{\text{Tx}}}_{\left(Bytes\right)}+{B^{\text{Rx}}}_{\left(Bytes\right)}, \end{array}$$

Where *P*^C^ and *P*^RF^ in Equation (3) are computation power and communication power (RF sending and RF receiving), respectively. The network usage δ in Equation (4) is computed as the sum of total bytes transmitted (*B*^Tx^) and received (*B*^Rx^) between a mobile device and the cloud. When a module, *m*_*i*_ ∈ *M*, executes on a mobile device, its communication power (${P^{\text{RF}}}_{i}$) and network usage (δ_*i*_) are both zero, and the battery power consumption (*P*_*i*_) will be equal to the computing power (*P*^C^). When a module, *m*_*i*_ ∈ *M*, is offloaded to the cloud for execution its computing power (${P^{\text{C}}}_{i}$) is zero, and the battery power consumption (*P*_*i*_) will be equal to the communication power (${P^{\text{RF}}}_{i}$). Therefore, we can write the expression in Equation (2) as,

(5) $$\begin{array}{l} min\left[\underset{m_{i}\in M}{\sum }\left(\alpha \left(m_{i}\right)\times \left({P^{\text{C}}}_{m_{i}},0\right)+\beta \left(m_{i}\right)\times \left({P^{\text{RF}}}_{m_{i}},\delta_{m_{i}}\right)\right)\right] \end{array}$$

Therefore, the effective partition of an MCH application can be obtained by optimizing the trade-off between the two objectives. This could be achieved by satisfying the expression in Equation (5).

## 4. Mobile-Cloud Hybrid Application Framework

A mobile-cloud application framework hybridizes the execution of tasks on mobile and the cloud endpoints. The framework provides the offloading API, which is used to convert the modules of mobile apps into offloadable. We implemented a simple MCH application framework (similar to the one used in Flores and Srirama, 2013) in Python for robotic tasks that are executed on a Linux-based systems. It uses a socket-based connection for code-offloading. The framework can be used for both offline and online profiling. In offline profiling, the framework takes the configurations as a command-line argument. It parses the configuration, and with a decision mechanism, it executes the mapped modules according to their respective digit's states in the configuration. In the online profiling, the framework is provided with the efficient configurations (previously found in offline profiling) and it switches between these configurations with a decision mechanism based on self-adaptivity and self-awareness and executes the modules according to their mapping in the configurations. Depending on the configuration level, a binary digit set as 1 indicates: (1) all the offloadable methods of the classes will be executed on the cloud (class-level), (2) the offloadable method of the classes will be executed on the cloud (method-level), (3) the offloadable classes and methods of other classes will be executed on the cloud.

### 4.1. Offline Profiling

In offline profiling, we instrument MCH applications to search for efficient configurations using multi-objective optimization. To achieve this, we have used a Python-based script, which uses an exhaustive search algorithm to iterates through all the possible configurations of an MCH application. As this work is targeting MCH applications created for battery-powered and Raspberry Pi controlled robots, the script automates the execution of the MCH applications on the robot. Moreover, we run the script on a PC, and it executes an MCH application (using a configuration) on the Raspberry Pi through *SSH*. Furthermore, the script records the total battery power consumption (i.e, Equation 3), network usage (i.e., Equation 4), and the total execution time of the application after one execution.

#### 4.1.1. Measuring Battery Power Consumption

**Figure 5:**
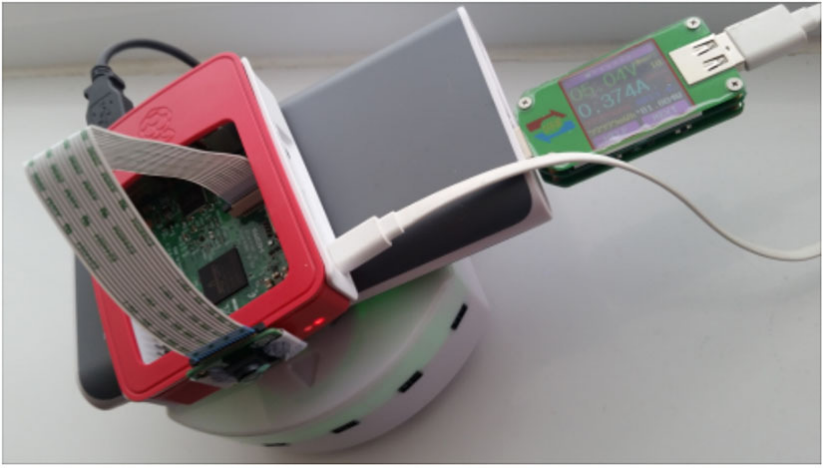
A battery-powered and Raspberry Pi controlled Themio-II robot. The robot performs a foraging task. A portable Anker Power-bank powers the Raspberry Pi. The Robot has its battery, which is charged from the Raspberry Pi through a USB data cable. A digital multimeter placed inline between the Power-bank and the Pi measures the power consumption of the Pi.

Battery power is consumed when an MCH application is executed on a battery-powered mobile device. Power is required for different components of the system to function, i.e., CPU, WiFi. To measure the power consumption of an application (as in Equation 3), there are platforms specific ways to use. In our previous work (Akbar and Lewis, 2017, 2018a), we have shown how to measure the power consumption for Android-based applications running on smartphones. In this work, we have targeted Python-based applications for battery-powered Raspberry Pi controlled robots. As the Pi models have no inbuilt current or voltage sensors that could be used for monitoring its current draw, or battery supply. Therefore, we have used a purpose-built digital multimeter, modeled UM24C, that is placed inline between a power-bank and the Pi, as shown in Figure 5. It can measure the power consumption of the Pi (per seconds) and is capable of sending the measurement via a Bluetooth connection to other devices. The Python-based script, running on a PC, receives these power measurements

#### 4.1.2. Measuring Network Usage

Network bandwidth is used when an MCH application executes its modules remotely on the cloud server endpoint. To measure the total network usage (as in Equation 4), there are platforms specific ways to use. In our previous work (Akbar and Lewis, 2017, 2018a), we used a built-in Android Library, *android.net.TrafficStats*, for recording data-sent (*B*^Tx^) and data-received (*B*^Rx^), for Android-based MCH applications. In this work, since we are targeting Python-based MCH applications for Raspberry Pi, we are using *T-shark* (a command-line network packet analyser tool) for measuring network usage. We have employed it in the Python-based script that run on a PC. The script captures data-sent *B*^Tx^) and data-received (*B*^Rx^) by an MCH application. After each run of the MCH application, the script records the total network bandwidth used.

### 4.2. Runtime Optimization of MCH Applications

We have carried-out the offline profiling in a controlled lab environment where a mobile device was operated in the best coverage location of the wireless base station (i.e., WiFi router). As there were no obstacles in the middle that could obstruct the WiFi signals, there was no wireless interference during the communication between the device and the cloud server. However, in the real-life operating environment, the external conditions change with time and can affect the application's execution. For example, a mobile device moves to a location where it is: (1) subject to wireless interference, or (2) receiving good signals but there is congestion in the network. In both scenarios, there will be packet loss during communication between the device and the cloud, which will result in network latency. The performance of applications will degrade when using code-offloading if the network has latency. By enabling the MCH framework with self-adaptivity and self-awareness makes it able to monitor its operative context to make run-time decisions, which is to use the right configuration and, therefore, avoid the network latency.

#### 4.2.1. Self-Adaptive MCH Application Framework for Dynamic Offloading

Self-adaptivity can enable MCH applications to modify their behavior at run-time, in response to the changing environment and make better decisions on how to use the available resources (Nakahara and Beder, 2018). In the context of MCH computing systems (i.e., smartphones and robots), as the mobile device moves, the WiFi signal level degrades over time. This is because of wireless interference caused by factors, such as obstacles in the middle of the communication channel between a mobile device and the base station. The signal degradation can cause packet loss, which results in network latency. The packet loss in the network (if high) can cause long socket-wait time plus TCP re-transmission at both endpoints (mobile and cloud).

In this work, we consider the WiFi signals as the changing environment, which changes across the network coverage area. To avoid network latency due to the low signal level, the MCH framework will monitor the signal levels continuously and based on which makes self-adaptive decisions at runtime. If the signals are good, the framework will switch to a configuration that is using code-offloading. If the mobile device moves to an area where the receiving signal level is bad, then by using the self-adaptive decision mechanism the framework will switch to the all-zero configuration keeping the computation on the device. For example, if the signal level is degraded (low) enough to cause latency due to packet loss, the framework will switch to run on such a configuration that does not use the code-offloading.

#### 4.2.2. Self-Aware MCH Application Framework for Dynamic Offloading

In line with the definitions from Lewis et al. (2011, 2016) and Kounev et al. (2017), we consider an MCH computing system to be self-aware when it gathers knowledge, not just about the environment, but about itself in that environment, on an ongoing basis. It is then able to use this knowledge to drive its decision making at runtime. In an MCH scenario, the availability of a high-quality network connection is one of the key requirements for the mobile device to make effective use of code offloading (Khan et al., 2015).

While a self-adaptive system observing the environment may base decisions on environmental factors, such as signal strength (as discussed above), self-awareness instead allows offloading decisions to be based on monitoring the device's behavior within that environment, specifically in this case, its success in communicating over the network. We operationalize this here by enabling the device to monitor the level of packet loss while running the code-offloading. When this is low, the self-aware MCH framework offloads code. When the packet loss is sufficiently high, then the code is run on the device. By observing the actual runtime impact of attempting to run the offloaded code, the device is no longer required to use estimates, based on externally observable proxy features (i.e., signal strength).

### 4.3. Online Profiling

We use online profiling to measure the battery power consumption, network usage and execution time of MCH applications, while the external environment is changing with time. The difference between offline and online profiling is that in offline profiling, we instrument the MCH applications to find efficient configurations. In the online profiling, the environmental keeps changes during the runtime and factors, such as network delay and congestion add latency and effect the code-offloading of MCH applications. Online profiling aims to validate the self-adaptive and self-aware decision mechanism of our framework.

For online profiling, we have created a controlled lab environment which can be used to instrument applications and keep changing the external operating environment. We will discuss the lab environment in section 6.

## 5. Scalable Mobile-Cloud Hybrid Applications

In this section, we will discuss search algorithms that could be used to find approximate to Pareto-optimal solutions and provide scalable options for MCH applications in terms of their size proportion to their offloadable modules. For a large number of offloadable modules, using the exhaustive search algorithm in offline profiling (to find Pareto-optimal configurations) is not a feasible option, regarding the time it takes to search the configuration sets. Therefore, depending on the time constraint, it may be suitable to get an approximation to the Pareto-optimal configurations in a reasonable amount of time. Multi-objective optimization (MOO) algorithms represent a viable alternative to potentially find this Pareto-optimal approximation set in one run.

### 5.1. Challenges in Scaling Up

To find the Pareto-optimal configurations, our approach is based on exhaustive search algorithm using the offline profiling, discussed in section 4.1. The exhaustive search enumerate all configurations to determine the energy-efficient configuration(s). While the exhaustive search is efficient in terms of time it takes for a small configuration set, it takes a large amount of time when the configuration set is large. As stated in Table 1, it would take too much time to search efficient configurations for an MCH application that have 20 offloadable modules. The total number of possible configurations increases exponentially (2^*n*^) when the number of offloadable modules, *n*, are increased for an MCH application. Such problems for which no known algorithm can find a solution to exact optimum in feasible amount of time are called NP-hard (Garey and Johnson, 1979). Therefore, to find Pareto-optimal configurations for MCH applications, exhaustive search algorithms are best to use for only a small configuration sets.

**Table 1:** The number of configurations increases exponentially when the offloadable modules increases.

**Modules**	**Configurations**
8	256
14	16,384
20	1,048,576 million

### 5.2. Approaches to Scale Up

#### 5.2.1. NSGA-II

NSGA-II, the Non-dominated Sorting Genetic Algorithm II, is a fast elitist population-based algorithm for Multi-objective Optimization (Deb et al., 2000). It has the following features:

1. It uses an elitist principle, i.e., the elites of a population are allowed to be carried to the next generation.
2. It uses an explicit diversity preserving mechanism (Crowding distance).
3. It emphasizes the non-dominated solutions.

For a comprehensive revision of the algorithm features, refer to Deb et al. (2000). Using the offline profiling (discussed in section 4.1), we implemented the NSGA-II algorithm in the Python script. For the NSGA-II implementation, we empirically chose some parameters based on results in the literature. The parameters were set as population size = 10, number of generation = 30 and the tournament population size = 5. The genetic parameters were set as crossover probability = 0.5 and mutation probability = 0.2.

#### 5.2.2. Two-Step Search Algorithm

The applications designed for mobile devices (smartphones, robots etc) use the available device-limited libraries or resources, such as GPS, sensors etc. Some classes that use them are not fit for offloading to the cloud as a whole. This results in a very few number of classes that are fit for offloading as a whole or partly. On the other hand, the methods in offloadable classes are usually in a high number. Therefore, the class-level configuration set in MCH applications, comparatively smaller than the method and hybrid-level, can be searched exhaustively in feasible time.

We design the Two-Step search algorithm to explore the configuration sets of MCH applications/tasks in two searching steps: (1) The first step aims to explore the class-level configuration set exhaustively to obtain elitist class-level configurations. (2) The second step aims first to search the collapsible method and hybrid-level configurations of the elitist ones and then randomly search their neighbor configurations up to some extent.

**Figure 6:**
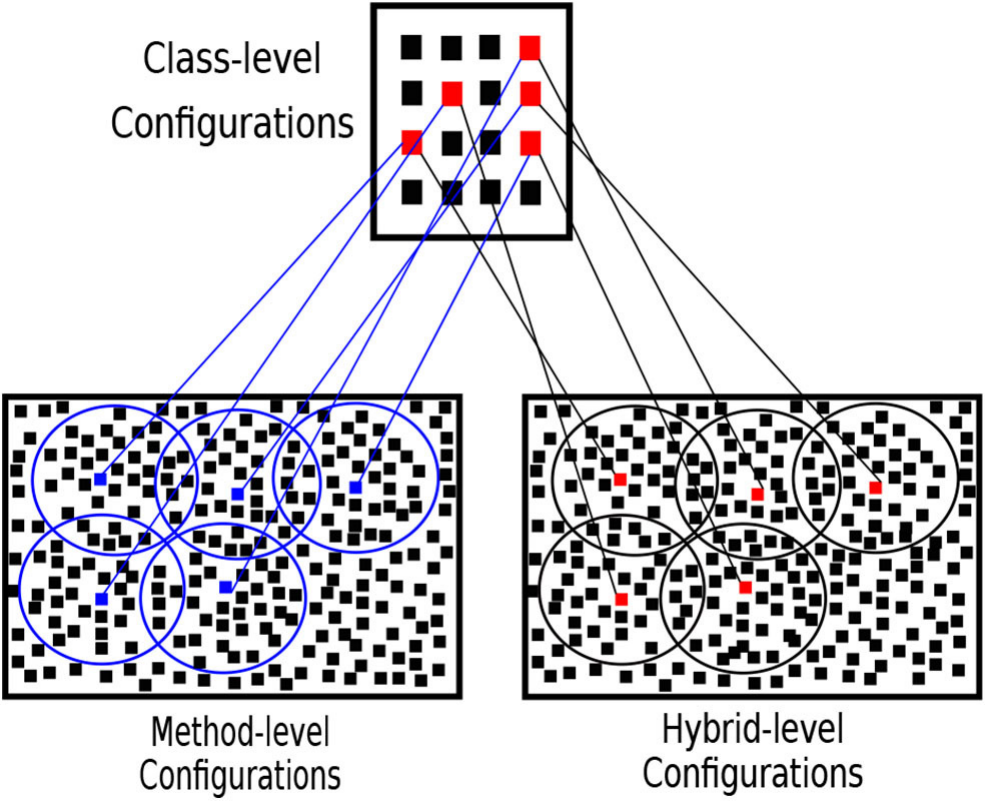
Two-Step search algorithm. The class, method and hybrid-level configuration sets are shown. The red dots in the class-level set represent the best configurations searched in first step. The blue dots in method-level and red dots in hybrid-level configuration sets are the collapsible configurations of the respective best class-level configurations. The circles represent the neighbor configurations of the collapsible best configurations the can be searched.

The best class-level configurations are the Pareto-optimal approximations. We create their collapsible method-level and hybrid-level configurations. The neighbor configurations are obtained by flipping the bits of the collapsible configurations randomly. Figure 6 illustrates how the Two-Step search algorithm works. The circles represents the limit of the neighbor search space.

Algorithm 1 presents the pseudocode for the Two-Step search. It starts by generating the class-level configurations, taking the number of class-level offloadable modules. In the first step, the efficiency of the generated class-level population is measured using the offline profiling. It finds the power and network usage by running the foraging task with the configurations. After the profiling is finished, it calculates fronts based on minimum power and network values of the configurations. The non-dominated solutions represent the Pareto-optimal approximation set.

**Algorithm 1:** Two-Step Search Algorithm

1: *pop* ← *Population*(*MODULES*) ⊳ First Step
2: profile(pop, “classLevel”)
3: pop.findfronts()
4: pop.findCrowdingdistance()
5: *elitePop* ← *pop*.*findClasslevelElitistPop*()
6: *methodLevelPop* ← *findMethodLevelPop*(*elitePop*) ⊳ Second Step
7: *hybridLevelPop* ← *findHybridLevelPop*(*elitePop*)
8: profile(methodLevelPop, “methodLevel”)
9: profile(hybridLevelPop, “hybridLevel”)
10: **procedure** profile(*pop, granularityLevel*)
11: *robot* ← *connectToDevice*()
12: **for each** *config* ∈ *pop*.*configs* **do**
13: *power, net, execTime* ← *getMeasurements*(*robot, config, granularityLevel*)
14: pop.saveMeasurements(config, power, net, execTime)
15: **end for**
16: **end procedure**

For selecting the elitist configurations from the class-level population, we apply the *crowding distance* (Deb et al., 2000) to rank the configurations. The selection of elitist configurations starts with the best non-dominated front and iterates through all fronts. A configuration presenting a high crowding distance value is selected. We select the best five configurations from the population as the elitist class-level configurations.

## 6. Experimental Study

To evaluate our proposed approach for battery-powered and Raspberry Pi controlled robots, we have used the work carried out by Heinerman et al. (2016) as a test-bed application. The application that they have created for performing a robotic task, called *foraging task*, is executed on a Raspberry Pi that is connected to a battery-powered *Thymio-2* robot (as shown in Figure 5). The robot collects red colored pucks in an arena and carry them to a blue colored target area in the corner of the arena. The controller of the robot is a feed-forward neural network, which evolves on-the-fly as it performs the task. In the code, they have implemented a Python-based library, NEAT (Stanley, 2004), to evolve an objective function that assesses the robot behavior for some time.

### 6.1. Application Modularization

The source code of the foraging task is available on Github^1^. After analysing the code, we found 4 classes having a total of 14 methods that are suitable for offloading. The offloadable modules do not use mobile-limited libraries or resources, i.e., sensors, GPS, LCD etc. We modularized the code and create configuration sets for the foraging task. For 4 classes (*n* = 4), the total number of class-level configurations are 16 (2^4^). For 14 methods (*n* = 14,) the total number of method-level configurations are 16, 384 (2^14^). For two classes and 14 methods, the total number of hybrid-level configurations are 16, 000. We have created a small Java-based tool^2^ that can create all possible hybrid-level configurations for an MCH applications by providing the number of classes and methods.

### 6.2. Executing the MCH Application

**Figure 7:**
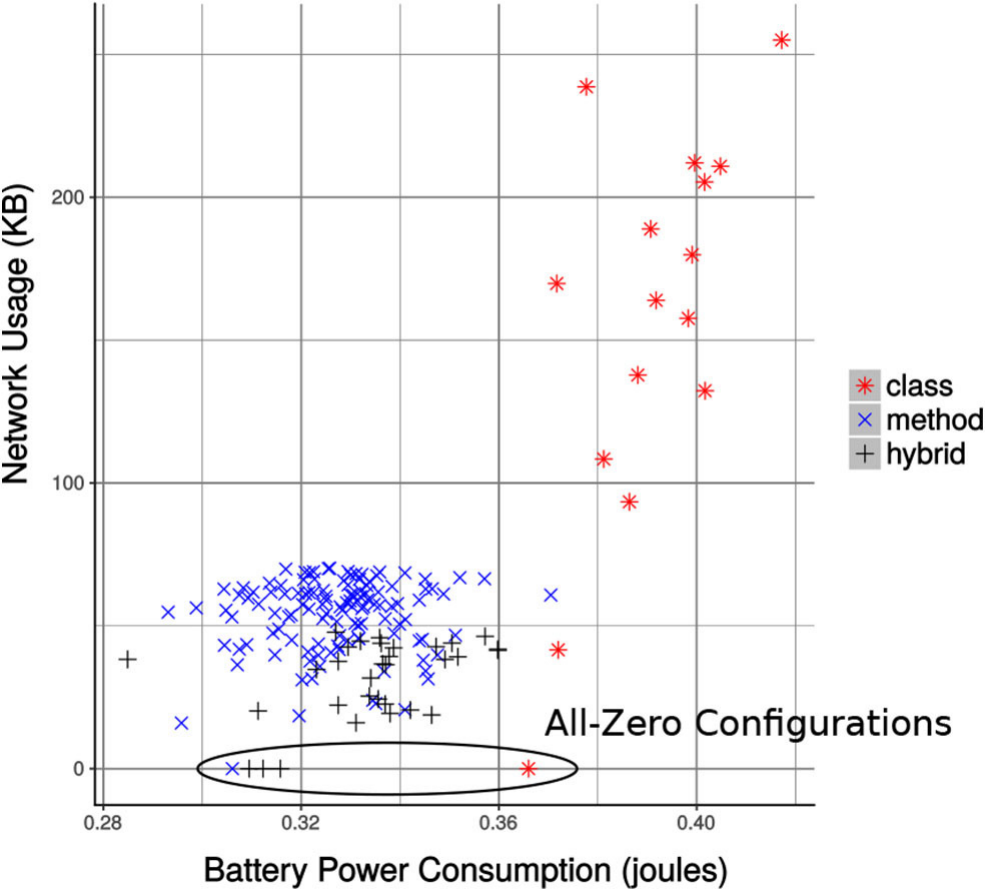
The configurations of all class-level and selected method and hybrid-level granularity for the MCH foraging task. All-zero configurations are those that run all the modules of the task only on the mobile device. Each dot on the plot represents the mean power and network usage of a configuration for 30 runs.

We have converted the foraging task to an MCH application and using the offline profiling (as discussed in section 4.1), we were able to execute it with all the configurations. The main goal of the offline profiling is to instrument the MCH application and measure the power and network usage while executing the application. However, due to some uncontrollable variables, it is necessary to perform any test over many algorithm runs. For example, the Raspberry Pi system was busy in doing system related tasks and the foraging task took more computation time or the data packets were dropping due to network congestion. Therefore, to eliminate the effect of the uncontrolled variables and obtain meaningful statistical significant results, we executed the Python-based script (discussed in section 4.1) multiple times. Doing so, we were able to take 30 samples of the 16 class-level configurations. For the method and hybrid-level configurations, initially, we executed all the configurations. We then select the best 150 configurations, residing near the Pareto-front. We took 29 more samples of the selected configurations, which results in a total of 30 samples. The mean power and network usage of these configurations are plotted and shown in Figure 7.

### 6.3. Finding Pareto-Optimal Configurations

To achieve an efficient MCH application, we employ a filter that only picks the non-dominated and statistically significant configurations. To achieve this, we created sets of collapsible configurations (as mentioned in section 3.3). We applied the filter on these collapsible sets to pick the non-dominated configuration(s) along with their statistically significant configuration(s) if they are present.

**Figure 8:**
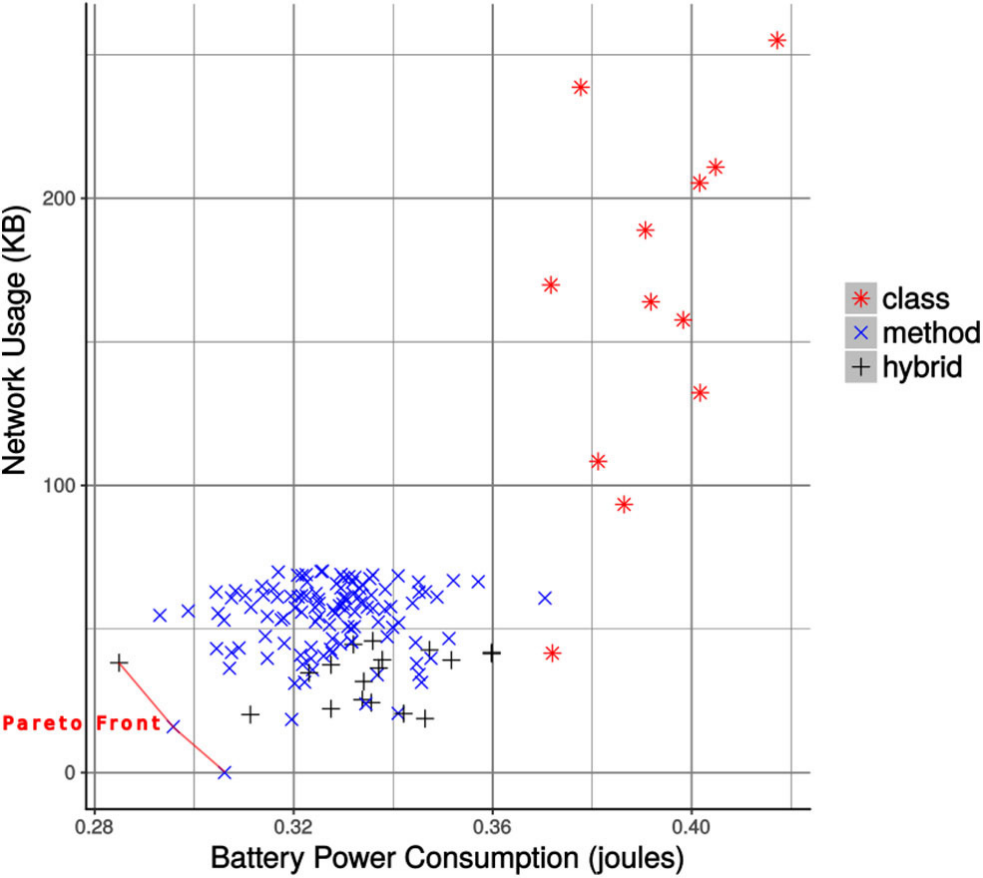
The filtered configurations of mixed granularity for the foraging task. The Pareto efficient configurations making the Pareto front are highlighted.

In the end, we created a final set of configurations by combining all the collapsible sets along with the configurations which were not collapsible. The non-dominated configurations in the final set are the Pareto-optimal configurations, listed in Table 2. These configurations are superior to the others when the objectives are considered (Srinivas and Deb, 1994). Also, these configurations optimize the efficiency trade-off and provide efficient alternatives to the MCH application in terms of battery power consumption and network bandwidth usage. The configurations from the final set are plotted in Figure 8, which also shows the Pareto front. As the offline profiling was carried out in a place near to the wireless base station with no interference or signal level degradation, the mean runtime of all the configurations were the same.

**Table 2:** Offline and online profiling of the mobile-cloud foraging task carried out by a Raspberry Pi controlled Thymio robot.

**Profiling**	**Configuration**	**Granularity level**	**Samples**	**Runtime (s)**	**Battery power consumption (J)**		**Network bandwidth usage (kB)**	
				**Mean**	**Mean**	**Standard deviation**	**Mean**	**Standard deviation**
Offline	C1 = 1011:001000000001	Hybrid	30	32.6	0.2849	0.0113	38.2503	0.0695
	C2 = 00100000000000	Method	30	32.6	0.2958	0.018	15.9743	0.0971
	C3 = 00000000000000	Method	30	32.6	0.3061	0.0136	0	0
Online (Scenario one)	C3	Method	1	251	0.4046	0	0	0
	C1	Hybrid	1	368	0.29	0	486.539	0
	Self-adaptive system (C1, C3)	Hybrid, Method	1	259	0.3793	0	192.117	0
	Self-aware system (C1, C3)	Hybrid, Method	1	264	0.3885	0	169.51	0
Online (Scenario two)	C3	Method	1	251	0.4046	0	0	0
	C1	Hybrid	1	415	0.4529	0	502.065	0
	Self-adaptive system (C1, C3)	Hybrid, Method	1	298	0.4232	0	312.917	0
	Self-aware system (C1, C3)	Hybrid, Method	1	255	0.3733	0	163.314	0

*The number of samples obtained of the configurations and their mean and standard deviation of battery power consumption and network bandwidth usage along with total samples and their average run time are stated*.

### 6.4. Runtime Decision Making of the MCH Framework

To evaluate the self-adaptive and self-aware approaches, our experimental work is based on online profiling of the MCH applications—foraging task. This will involve measuring the performance of both approaches in terms of minimizing the two objectives and comparing it with static offloading and no offloading. We created a lab-based controlled environment in which the robot performs the foraging task and a Python-based script simulates the change in the signal level over time. We considered the signal level variation, caused by an interference, as the changing environment during the course of performing the foraging task. One cycle of signal level variation takes 60 s. The cycle starts from a signal level of −32*dBm* and decreases until −90*dBm*. It then starts increasing back until −32*dBm* and then repeats itself. As the signal level degrades it causes packet loss. We imposed the packet loss at the Linux kernel-level on the cloud side, which is aligned with the change in the signal level. For this to achieve, we used *tc* and *Netem*^3^ command-line tools to randomly drop the number of packets. As shown in **Figure 10**, we modeled the packet loss with respect to the signal level as: (1) from −32 to −70*dBm* the packet loss is 0%, (2) from −71 to −80*dBm* the packet loss is 20%, (3) from −81 to −85*dBm* the packet loss is 50% and lastly (4) from −86 to −90*dBm* the packet loss is 80%. We estimate these measurements by using *Wireshark* to observe the packet loss concerning the signal level while moving the robot in the network coverage area.

#### 6.4.1. Profiling the Pareto-Optimal Configurations at Different Signal Levels

**Figure 9:**
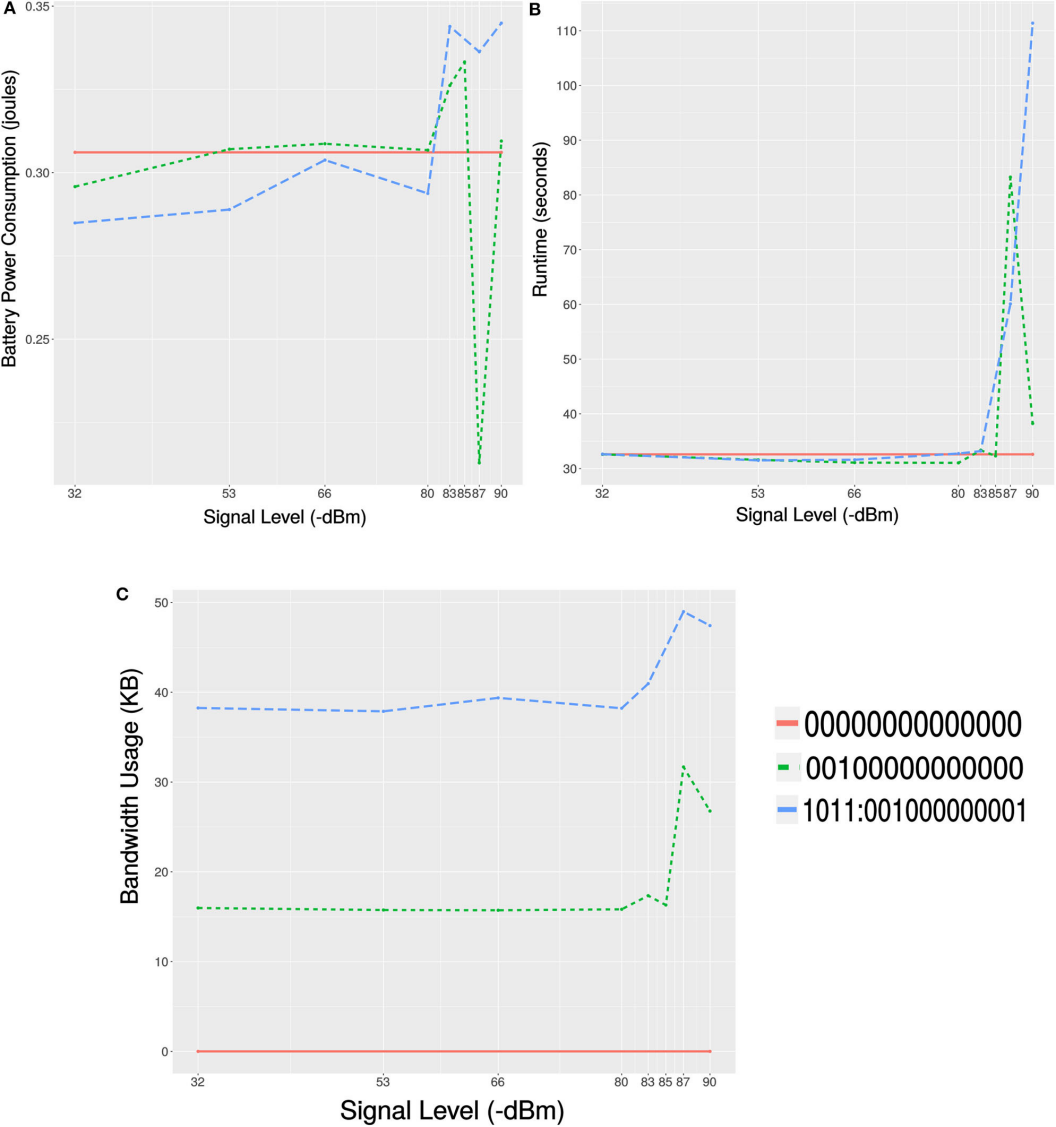
Plots showing the results of profiling the Pareto-optimal configurations at different signal levels. **(A)** Mean power consumption of 30 samples at different signal levels. **(B)** Mean execution time of 30 samples at different signal levels. **(C)** Mean network bandwidth usage of 30 samples at different signal levels.

The offline profiling (discussed in section 4.1) was carried-out while keeping the robot under the footprint of good signal level (around −32*dBm*) from the wireless access point. As the robot moves around the network coverage area, the signal level changes and in some cases might degrade enough to cause large amount of packet loss. For the self-adaptive decision mechanism to switch to the all-zero configuration (C3), we need to find the signal level threshold. To find the signal level threshold for the self-adaptive switching, we did profiling of the task where the robot was moving in the network coverage area and the signal level was changing. While measuring the power consumption, network usage and execution time, we executed the foraging task for 30 times at various signal levels ranging from −53 to −90*dBm*. In these runs, we recorded the efficiency of the two configurations (*C*1 and *C*2) that use offloading at 7 different signal levels. Configuration *C*3 was kept same as before at all levels as it does not use offloading. The average battery power consumption, execution time and network usage at these different signal levels are recorded and shown in Figures 9A–C, respectively.

#### 6.4.2. Determining the Threshold for Self-Adaptive Switching

We can see in Figure 9A that the battery power consumption in case of configurations *C*1 and *C*2 changes with respect to a change in the signal level. The signal degrades from a better (−32*dBm*) to a poor (−80*dBm*) level, during which *C*1 performed better than *C*2 and *C*3. Below the signal level of −80*dBm* is an unstable network zone. In this zone, the power consumption can either increase very high (due to TCP retransmission) or decrease very low (due to Latency). As shown in Figure 9B, with the configuration *C*2 the execution suffered from a long socket-wait (latency) at signal level −87*dBm* (unstable zone). While the mobile was suffering from packet loss at the receiving end the cloud was continuously retransmitting packets and using more network, as shown in Figure 9C. Similarly, execution with *C*1 suffered from TCP retransmission at the signal level of −83*dBm* in the unstable zone. This caused high delay and high network usage as shown in the Figures 9B,C, respectively. Based on the above analysis, we choose the threshold for the self-adaptive switching at a signal level of −80*dBm*. As shown in Figure 9A, *C*1 performed better above −80*dBm* and *C*3 performed better below −80*dBm* in terms of battery power consumption. Therefore, the self-adaptive decision mechanism of the framework while executing the MCH foraging task will use *C*1 on and above −80*dBm*, and will switch to *C*3 when the signal level is below (−80*dBm*).

#### 6.4.3. Online Profiling: Foraging Task

The rate of packet loss increases due to factors like wireless interference, link failure and network congestion. The packet loss causes latency in the network and, therefore, can affect the execution of the MCH application if the mode of static code-offloading is being used. As a result, at the mobile endpoint, long socket-wait time will cause more delay to the completion of the task. At the cloud, long socket-wait will cause the mobile to retransmit the TCP packets with the cost of using more battery power and network usage. We consider the following two scenarios, which are intended to capture two contrasting types of environment that might be encountered by a robot.

##### 6.4.3.1. Scenario 1

**Figure 10:**
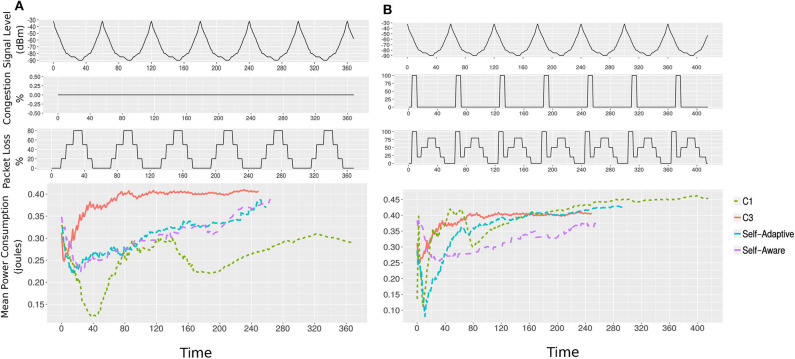
Plots showing results of the two scenarios. Mean of battery power consumption of configurations *C*1, *C*3, self-adaptive, and self-aware approaches with a passage of time are plotted. The lines end show the completion time. The packet loss caused by signal level degradation and congestion is also shown. **(A)** Scenario one: the self-adaptive and self-aware MCH tasks both achieve better optimization in a changing environment (signal level variation). **(B)** Scenario two: With a packet loss caused by signal level and network congestion the self-aware approach out-performed the self-adaptive, *C*1 and *C*3 in terms of using less power and network bandwidth. It is due to the fact that the robot monitored the packet loss by observing its runtime impact, i.e., avoiding latency.

In the first scenario, we will consider zero per cent congestion in the network. As shown in Figure 10A, the congestion is kept zero during the course of the task execution.

##### 6.4.3.2. Scenario 2

In the second scenario, we introduce a high degree of network congestion (100%), where the packet loss is 100%, as shown in Figure 10B. Adding the congestion simulates the behavior of the unstable zone, discussed in section 6.4.2. During the congestion, we assume that the self-adaptive approach will likely be unable to switch to *C*3 as the signal level would still be good (from −50 to −70*dBm*).

#### 6.4.4. Results and Analysis

To determine the performance of the self-adaptive and self-aware enabled framework, we executed the MCH foraging task for an increased time duration. Doing the online profiling and using the two scenarios, we measured the mean power consumption, bandwidth usage and run-time of the task with *C*1, *C*3, and enabling either the self-adaptivity or self-awareness approach. The measurements are listed in Table 2. The execution of the task with *C*3, which does not use code-offloading, is used for both scenarios. The executions of the task with *C*1, which uses code-offloading, suffered from high latency in both scenarios.

##### 6.4.4.1. Scenario 1

In scenario one, the execution of the task with the self-adaptive or self-aware approach, which switches between *C*1 and *C*3 according to the signal level (self-adaptivity) and packet loss (self-aware), consumed less battery power than *C*3 with the cost of using network bandwidth. The executions did not suffer from latency, as happened in case of *C*1, because the framework switches to all-zero configuration (*C*3) when the signal level is low and causing packet loss, as shown in Figure 10A.

##### 6.4.4.2. Scenario 2

In scenario two, the execution of the task with the self-adaptive or self-aware approach again performed better than *C*1 by taking less time to complete. However, with the self-adaptive approach, the execution suffered from latency due to packet loss caused by the high network congestion. The task execution with the self-aware approach performed better by avoiding the packet loss caused by both network congestion and low signal level. The self-aware MCH framework used less battery power than *C*3 and less network bandwidth than *C*1 and self-adaptive, and also finished in good time as shown in Figure 10B.

### 6.5. Scalable MCH Application—Robotic Foraging Task

We will compare the outcomes of the two multi-objective optimization algorithms (NSGA-II and Two-Step) discussed in sections 5.2.1 and 5.2.2, respectively. Performance assessment include both the quality of the outcome as well as the computational resources needed to generate this outcome. Concerning the latter aspect, the number of fitness evaluation will be the same for both algorithms. As to the quality aspect, comparing solutions in the presence of multiple criteria, the Pareto dominance concept must be used. However, when comparing two sets of solutions, some solutions in either set can be dominated by solutions in the other set and some solutions can be incomparable. As both algorithms use random optimization methods, due to the stochastic nature of the algorithms, for obtaining a well-based judgement related to the quality performance, it is necessary to perform any test over many algorithm runs. Therefore, to eliminate the random effect and obtain meaningful statistical significant results, we executed both algorithms 10 times, each time with a different seed for random number generator for both method and hybrid-level configuration sets. The number of objective function evaluation was kept the same (300) for both algorithms in each run. To compare the quality of the solution sets produced by these two MOO algorithms, we used two different unary indicators: (1) Hypervolume Indicator (S-metric) and (2) Attainment Surface. These indicators are discussed in the following sub-sections.

#### 6.5.1. Hypervolume Indicator

**Figure 11:**
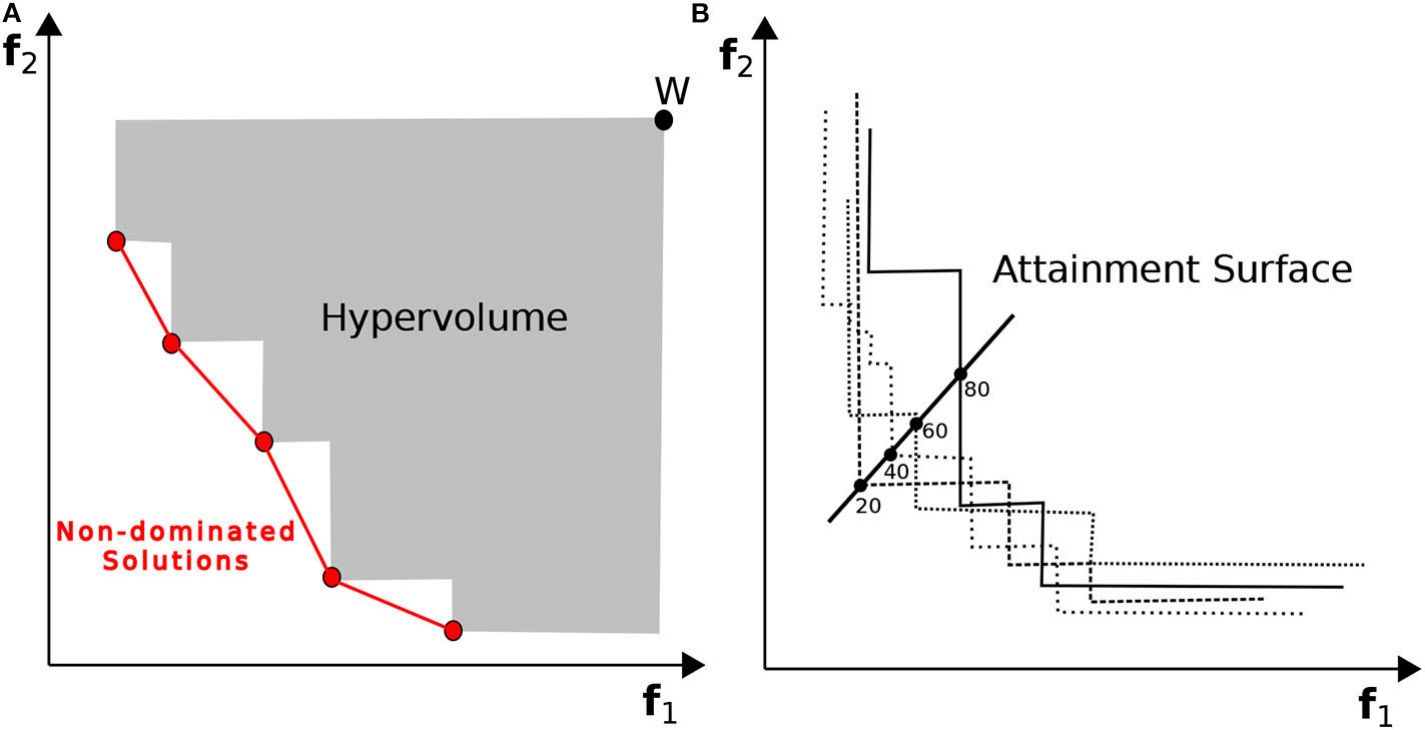
In **(A)** the hypervolume for a minimization problem, calculated as the area enclosed by the non-dominated solutions and a chosen reference point (w), is shown. It computes the size of the region that the non-dominated points dominates. In **(B)** the attainment surface is created for several non-dominated solutions for a minimization problem. It describes the distribution of the obtained non-dominated set using the notion of goal-attainment.

The hypervolume is a unary value, which is calculated as the sum of the areas formed by points on the non-dominated front and a chosen reference point (w). Figure 11A shows the area of the bi-dimensional region enclosed by a set of non-dominated points and a reference point (W) considering a minimization problem. It is a well-known quality measure in evolutionary multi-objective optimization to evaluate the performance of search algorithms (Auger et al., 2009; Bradstreet, 2011). Hypervolume takes into account the diversity as well as the convergence of the non-dominated solutions. The reference point (w) represents some upper boundary of the region within all feasible points lie. The basic idea is that, for a bi-dimensional minimization problem, the larger the area dominated by one non-dominated set in the objective space, the better the set is. To compare the performance of the two MOO algorithms, we will calculate the hypervolume of the obtained Pareto-optimal approximation set for each algorithm after each run.

#### 6.5.2. Attainment Surface

The attainment surface corresponds to a region in the objective space which is attained by (dominated by or equal to) the good solution returned by a MOO algorithm. It is formalized in the concept of k%-attainment surface. Figure 11B shows the results obtained from an arbitrary multiple runs of a MOO algorithm describing the distribution of the obtained non-dominated set using the notion of goal-attainment. The best attainment surface is the limit between the region attained by at least one run and the objective vectors never attained by any run. Whereas, the worst attainment surface delimits the region attained by all runs. The graphical visualization of attainment surface is a powerful tool providing a good insight of the algorithm performance. López-Ibáñez et al. (2010) developed graphical tools for the analysis of bi-objective optimization algorithms that plot the attainment surface of the solution sets. We will use the tool in *R* to plot the probabilistic distribution of the configurations along the Pareto front, obtained by the two algorithms. The tool calculates the empirical attainment function (EAF), which provides a summary of the outcomes of the 10 different runs of each algorithm. By plotting and comparing the EAFs of the two algorithms, we will be able to pin-point several performance behaviors.

#### 6.5.3. Hypervolume Indicator for the MCH Foraging Task

**Figure 12:**
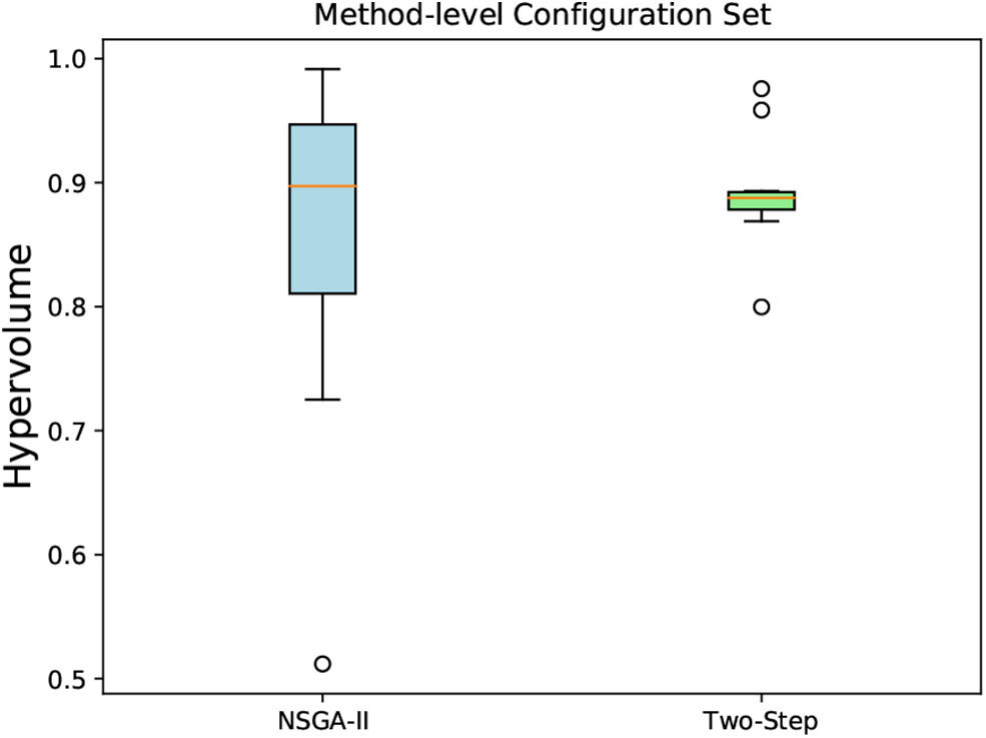
A box-and-whisker plot showing the hypervolume distribution of 10 independent runs of NSGA-II and Two-Step MOO algorithms. The non-dominated configurations obtained by the algorithms were for the method-level configuration sets created for the MCH foraging task.

**Figure 13:**
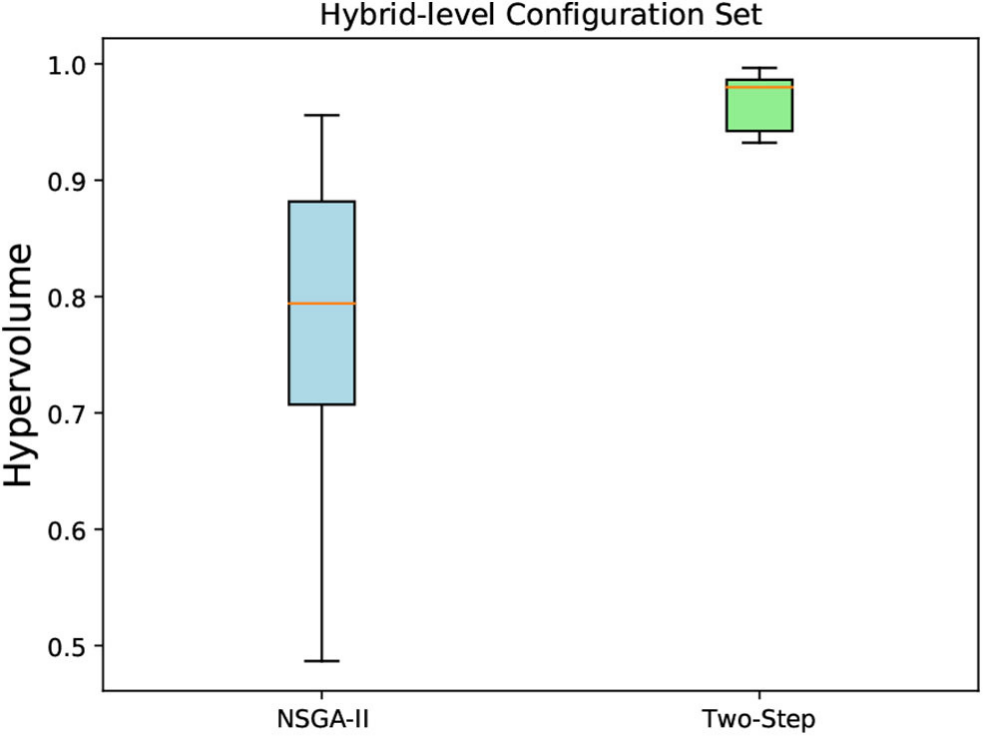
A box-and-whisker plot showing the hypervolume distribution of 10 independent runs of NSGA-II and Two-Step MOO algorithms. The non-dominated configurations obtained by the algorithms were for the hybrid-level configuration sets created for the MCH foraging task.

We run each algorithm 10 times, and the hypervolume was calculated for each obtained non-dominated solutions for both algorithms. The reference point used was the same in all obtained solutions in both algorithms. The box-and-whisker plots in Figures 12, 13 show the distribution of hypervolume values for the method-level and hybrid-level configuration sets, respectively. For the method-level configuration set, the medians of the hypervolume for both algorithms are nearly at the same level. However, the underlying distributions are very distinct. It indicates a better consistency in the hypervolume values (for the 10 runs) for the Two-Step algorithm when compared with NSGA-II. Given the amount of time (2 weeks) that both algorithms took to generate these results, we can say that the Two-Step algorithm performed well. If the time constraint is removed, the NSGA-II might perform better by increasing its number of generations.

**Figure 14:**
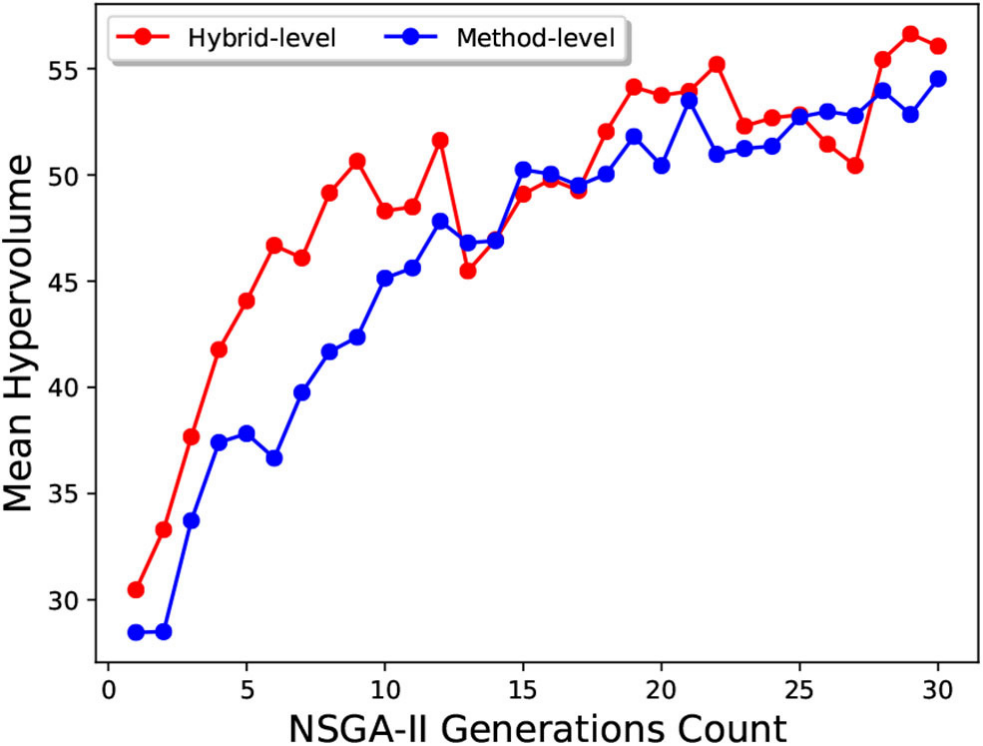
The mean hypervolume obtained for 10 runs of NSGA-II throughout the generations. The dots represent the mean hypervolume level of the 10 runs with respect to the number of generations. The two lines are for method-level and hybrid-level configuration sets created for the MCH foraging task.

As we can see in Figure 14, the mean hypervolume value, corresponding to the mean hypervolume value for the 10 runs throughout the generations, has not fully converged for the method-level configuration set. For the hybrid-level configuration set, it is evident from Figure 13 that the Two-Step algorithm performed better than the NSGA-II. In case of NSGA-II, the distribution of the hypervolume values shows a large spread. Similar to the method-level configuration set, the mean hypervolume value has not fully converged as shown in Figure 14.

#### 6.5.4. Attainment Indicator for the MCH Foraging Task

**Figure 15:**
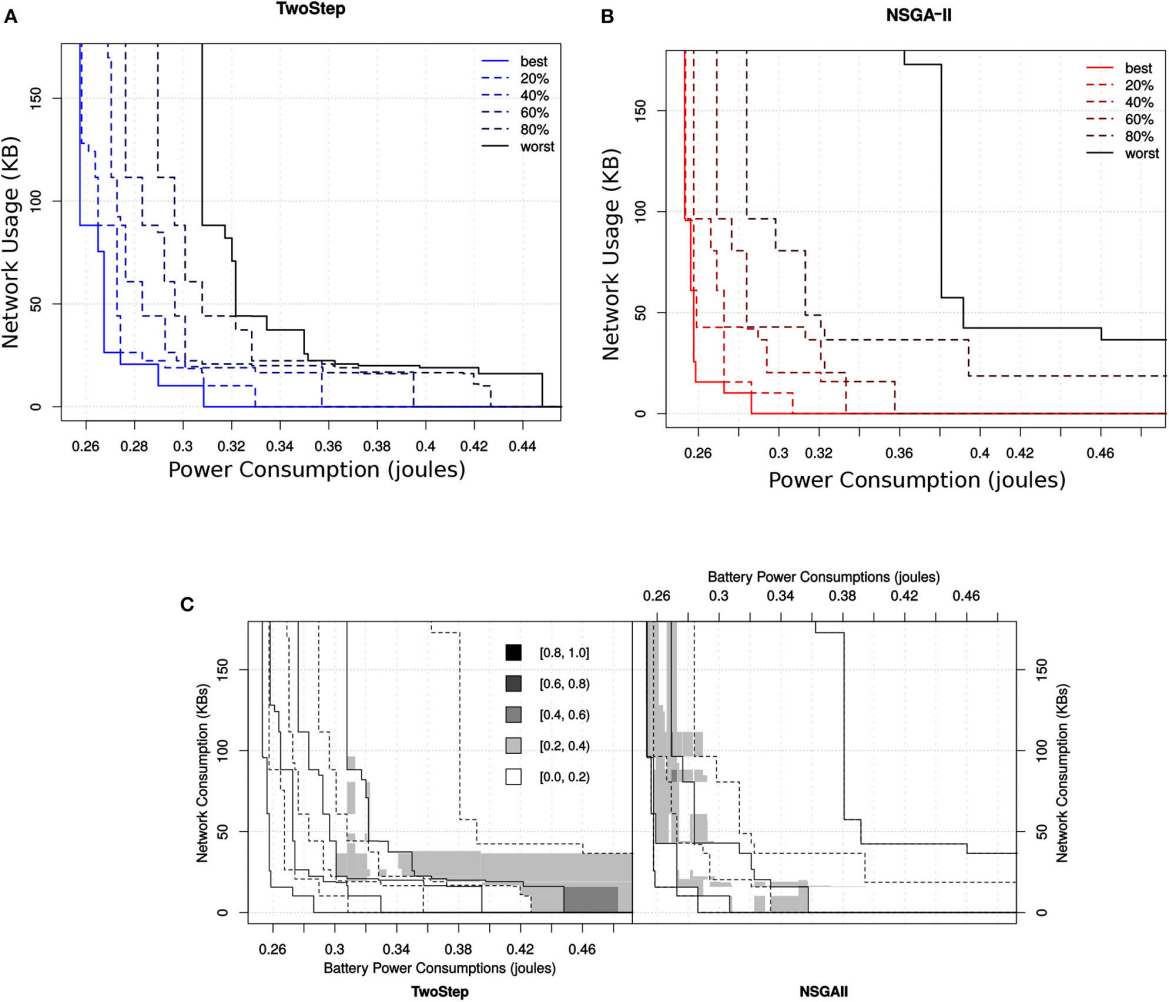
Ten independent outcomes obtained for the method-level configuration set by the two different MOO algorithms. In **(A)** the attainment surface of Two-Step is shown with respect to six quartiles. In **(B)** the attainment surface of NSGA-II is shown with respect to six quartiles. In **(C)** the location of the difference between the EAFs of the two algorithms is shown where the gray level represents the magnitude of the difference.

To visualize the behavior of the two algorithms and illustrate where in the objectives space and by how much the outcomes differ for the method-level and hybrid-level configuration sets, we plotted the attainment surface of the non-dominated solutions obtained by the two algorithms. In Figures 15A,B, we can see the attainment surface of the obtained non-dominated method-level configurations by NSGA-II and Two-Step search algorithm, respectively. Additional to the best and the worst attained surface, we have also shown the attainment surface with other percentiles (20, 40, 60, 80%). We can see that the attainment surface of method-level configurations obtained by the Two-Step search algorithm is more compact with respect to all percentiles.

**Figure 16:**
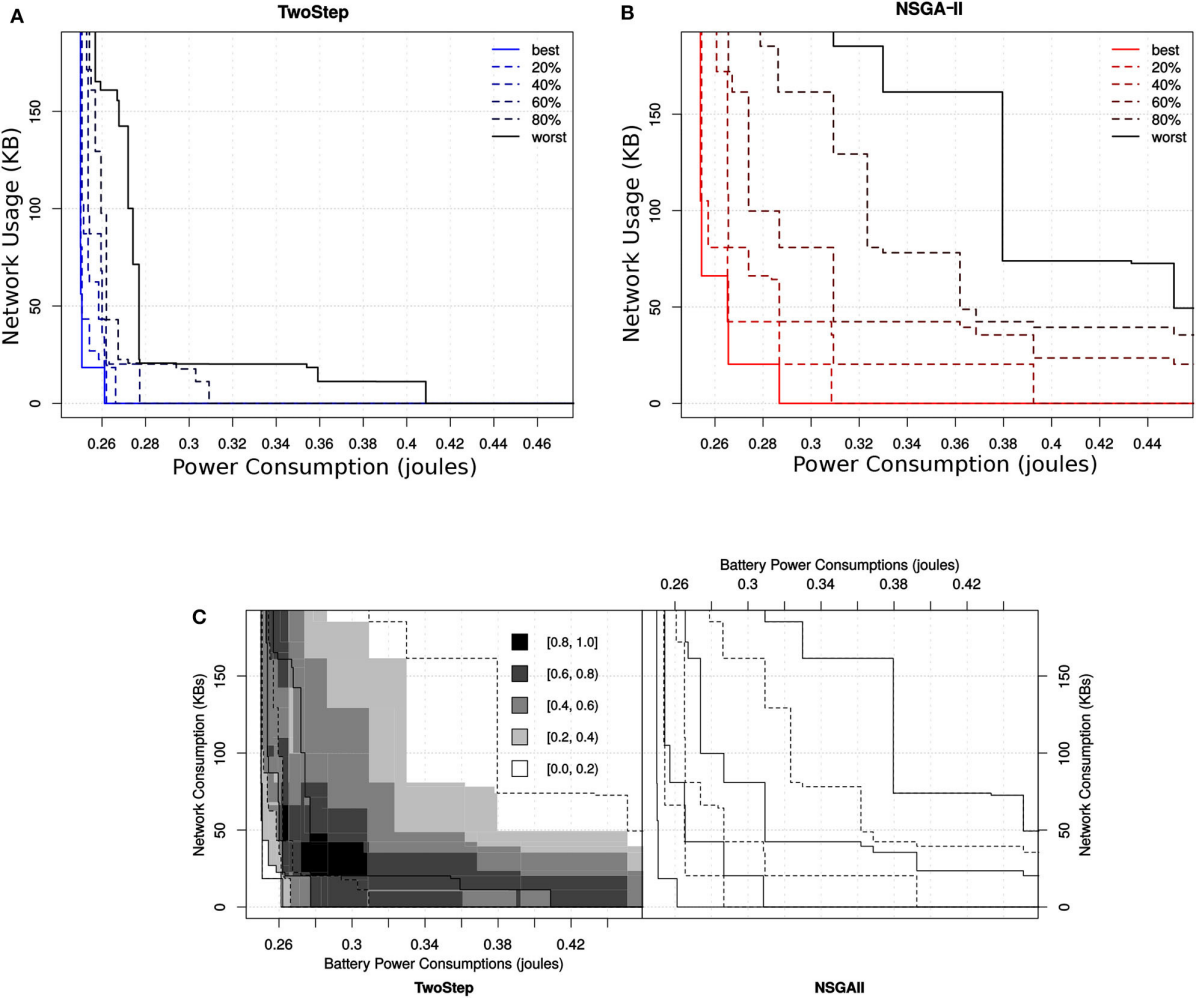
Ten independent outcomes obtained for the hybrid-level configuration set by the two different MOO algorithms. In **(A)** the attainment surface of Two-Step is shown with respect to six quartiles. In **(B)** the attainment surface of NSGA-II is shown with respect to six quartiles. In **(C)** the location of the difference between the EAFs of the two algorithms is shown where the gray level represents the magnitude of the difference.

Similarly, in case of hybrid-level configurations, the attainment surface obtained by the Two-Step search algorithm is even more compact with respect to all percentiles as shown in Figure 16. Figure 15C points the differences between the two algorithms with respect to their corresponding EAFs. The value of the EAF indicates the probability of attaining an area in the objective space. The performance of an algorithm would be considered better than the other if its EAF value at certain area is larger than the other. The gray level represents the magnitude of the difference. From the figure, we can see that the Two-Step search algorithm performs better in terms of finding good solutions toward minimization of network usage that were not good toward the minimization of power usage. On the other hand, the NSGA-II performs better toward high quality configurations for the minimization of power consumption and also slightly for the minimization of both objective.

#### 6.5.5. Results and Analysis

The above analysis of the obtained results provide some key facts regarding how much an MCH application can be scalable, proportional to the number of their offloadable modules, to produce efficient configurations that optimize the trade-off between power and network usage.

##### 6.5.5.1. Small-scale MCH applications

According to our experiments, offloadable modules between 2 and 8 would lie the so-called small-scale MCH applications. For a small-scale MCH applications, the exhaustive search algorithm is appropriate to use for finding the Pareto-optimal configurations. In the Two-Step search algorithm, we used an exhaustive search to find Pareto-optimal class-level configurations (in the first step) because of a small number (4) of class-level modules. The exhaustive search completed in around 30 min for the class-level configuration set of cardinality equals to 16. However, for the total number of configurations (32, 400) in all the three sets (class-level, method-level and hybrid-level) combined, the exhaustive search took more than a month to find the Pareto-optimal configurations using the offline profiling (discussed in section 4.1).

##### 6.5.5.2. Medium-scale MCH applications

According to our experiments, offloadable class-level modules < 8 and method-level modules higher than 8 would lie the so-called medium-scale MCH applications. For a medium-scale MCH applications, the exhaustive search algorithm will take a large amount of time to complete and, therefore, would not be practical to use. In this case, Evolutionary Algorithms, such as NSGA-II, are appropriate to use, which can approximate the Pareto-front solutions in a reasonable amount of time. For the Foraging task, where the number of class-level configurations is equal to 16 and the combined method-level and hybrid-level configurations are equal to 32, 384, both the Two-Step and NSGA-II were feasible to use. They took about 2 weeks time for the 10 independent runs to complete.

##### 6.5.5.3. Large-scale MCH applications

According to our experiments, offloadable class-level and method-level modules higher than 8 would lie the so-called large-scale MCH applications. For a large-scale MCH applications, the Two-Step search algorithm would not be practical to use. For such applications, finding a scalable solution is an open research question that we will leave for the future work.

## 7. Conclusion

In this paper, we presented a self-aware and scalable solution to efficient MCH robotic tasks. Using multi-objective optimization and code-offloading techniques, we develop an MCH application framework. We presented a workflow to modularize the source code of applications developed for mobile devices and based on the three granularity level we create class-level (coarse-grained), method-level (fine-grained) and hybrid-level configurations for the task. By doing offline profiling and using the framework, MCH robotic tasks can be executed with the configurations and the battery power consumption and network usage are measured at runtime to find efficient configurations. For scalability, we presented a Two-Step search algorithm that can find the efficient configurations in reasonable amount of time. A self-aware and self-adaptive decision mechanism of the framework can use the efficient configurations in the fly to optimize the tradeoff between battery power consumption and network usage.

We evaluate the technique using a battery powered and Raspberry Pi controlled Thymio Robot performing an MCH foraging task. An exhaustive search algorithm, which took more than a month, finds Pareto-optimal configurations that optimize the tradeoff between power consumption and network usage. We used these configurations to evaluate self-adaptive and self-aware decision mechanism for which we carried out experiments using two different scenarios. In scenario one, we created a lab-based controlled environment in which a change of network signal level would cause packet loss, resulting in TCP socket-wait delay or packet retransmission. In scenario two, in addition to the poor signal level, network congestion due to overloading would also cause packet loss.

The self-adaptive switching was based on an evaluated signal level threshold (−80*dBm*). The robot performing the task would switch between two Pareto-optimal configurations, one which would execute all modules on the robot when the signal level was below the threshold, other which would use code-offloading when the signal level was above the threshold. The self-aware decision, however, was based on monitoring the packet loss by the robot within itself rather the change of the signal level. With high amount of packet loss the task would switch to the Pareto-optimal configuration that would execute all modules on the robot and with zero to small amount of packet loss the task would switch to the configuration that use code-offloading. The self-adaptive and self-aware switching were implemented to avoid network latency and to minimize the power and network usage. The experimental results indicate that in scenario one, using the self-adaptive and self-aware decisions performed better than using static offloading and no offloading. In scenario two, the self-aware decision mechanism was better than the self-adaptive, static offloading and no offloading. As the robot was monitoring the packet loss by observing its runtime impact it avoid the latency caused by network congestion.

To make the framework scalable, in terms of finding non-dominated solutions in a large configuration set, we developed a Two-Step search algorithm. We carried out an experimental study to compare the outcome of the Two-Step search algorithm with NSGA-II. The assessment was based on the quality of the outcome of the algorithms. Due to the stochastic nature of the algorithms, to eliminate the random effect and obtain meaningful statistical significant results, we executed both algorithms 10 times, each time with a different seed for random number generator for both method and hybrid-level configuration sets. We used two performance indicators: the Hypervolume Indicator and visualization of the Attainment surface. The results shows that the Two-Step algorithm performed well, given the amount of time (2 weeks) that both algorithms took to generate the results.

## 8. Future Work

In order to validate the idea of optimizing the battery power consumption and network usage in MCH robotic tasks, we presented a solution that with a self-aware decision mechanism can optimizes the efficiency trade-off and is scalable for a small to medium-scale robotic tasks. In the future work, we plan to apply this technique to large scale MCH robotic tasks, as well as applications created for smartphones/tablets. Further, we plan to provide a formal method for self-adaptive and self-aware decision mechanisms and also evaluate the framework with other context factors, such as resource usage, code type, cloud-side context and using non-WIFI networks (e.g., LoRa/LoRaWAN, NB-IoT, SigFox, etc.).

## Data Availability Statement

The datasets generated for this study are available on request to the corresponding author.

## Author Contributions

All authors listed have made a substantial, direct and intellectual contribution to the work, and approved it for publication.

## Conflict of Interest

The authors declare that the research was conducted in the absence of any commercial or financial relationships that could be construed as a potential conflict of interest.

## Footnotes

^1^https://github.com/jvheinerman/NEATThymio

^2^https://github.com/aamirakbar/Creating-Hybrid-Level-Configurations-for-MC-hybrid-Apps

^3^https://wiki.linuxfoundation.org/networking/netem
